# The Analgesic Museum

**DOI:** 10.3389/fpain.2022.1019632

**Published:** 2022-10-21

**Authors:** Ian J. Koebner, Bethney Bonilla, Jenny Slatman, Manon Parry

**Affiliations:** ^1^ Anesthesiology and Pain Medicine, University of California, Davis, Sacramento, CA, United States; ^2^ Romance Languages and Literatures, Harvard University, Cambridge, MA, United States; ^3^ Center for Healthcare Policy and Research, University of California, Davis, Sacramento, CA, United States; ^4^ Culture Studies, Tilburg University, Tilburg, Netherlands; ^5^ American Studies and Public History, University of Amsterdam, Amsterdam, Netherlands; ^6^ Medical History, Vrije Universiteit, Amsterdam, Netherlands

**Keywords:** persistent pain, museums, social connection, art, wellness

## Abstract

This manuscript uses the perspectives and insights that emerged from the Analgesic Museum conference held virtually on March 11, 2022 as a mechanism for considering the role museums and artists can play in the public health effort to reduce the burden of persistent pain. One hundred and fifty-seven individuals from 22 countries registered for the Analgesic Museum conference. The event explored the intersection of art and pain management practices with presentations centered on three domains of interest: exhibition development, arts experiences and practices, and research and creative scholarship.

## Introduction

Persistent pain affects hundreds of millions of individuals globally with a recent review in the Lancet boldly proclaiming, “it is difficult to overestimate the burden of chronic pain.” (1) While social factors, such as social isolation and loneliness, can greatly impact the lived experience of pain, the biomedical community has not operationalized many treatment approaches to address these factors (2). Concurrently, a meaningful body of scientific literature now supports what practitioners of the humanities have argued for centuries—the arts can improve health (3). But can the arts relieve pain?

On March 11, 2022, 13 speakers along with 157 registrants from 22 countries came together on a virtual platform to explore the potential of cultural engagement in museums and art spaces to reduce the burden of pain. The day-long conference titled, *The Analgesic Museum* focused on three overlapping areas of interest:

• **Exhibition development** to showcase the aesthetics of analgesia
• **Arts experiences and practices** to reduce the burden of persistent pain
• **Research and creative scholarship** to explore how museum-based interventions can lessen pain

The goal of the conference was to seed an international interdisciplinary network of scientists, museum and healthcare professionals, individuals living with persistent pain, and artists committed to investigating the aesthetics and impact of museum engagement to reduce the burden of persistent pain. To provide highlights from the day with a broader audience this paper offers summaries of the featured speakers' perspectives and insights. The entire conference can be viewed for free at: https://health.ucdavis.edu/pain/acupuncture/CrockerArtRx.html.

## Setting the scene

**Christopher Bailey**, Arts and Health Lead for the World Health Organization (WHO), and **Melissa Menzer**, Senior Program Analyst at the National Endowment for the Arts (NEA), began the conference by helping to frame, through both personal testimonial and review of the evidence base, the impact the arts have on health and wellbeing. A WHO review on the role of the arts in improving health and well-being, which included over 3,000 studies, identified a major role for the arts in preventing ill health and promoting wellbeing across the lifespan (3). Relevant to this conference the review found that little work has been done in the art and health field focused on persistent pain, a finding that has been confirmed elsewhere in the literature (4). Menzer detailed the NEA's recent review on arts strategies for addressing pain and the opioid crisis. This landmark review produced several findings that informed both the intention and structure of the Analgesic Museum conference. First, the NEA review found that the majority of studies conducted on pain management focused on post-operative pain (vs. persistent pain) and investigated music-based interventions (vs. museum-based interventions) (5). Second, the report called for more research on the arts’ impact on persistent pain and on the social dimensions of pain (5). The conference responds to these findings by not only highlighting projects that specifically examine the role of non-music-based arts programming to address persistent pain, but through the identification of topic areas—Exhibition development; Arts experiences and practices; Research and creative scholarship—that will foster more research and programming in these gap areas of study and practice.

## Exhibition development

### How do we curate art to reduce the burden of pain?

**Sabrina Kamstra**, Chief Curator and Head of the Art Department at the Amsterdam University Medical Center (UMC) oversees a collection of more than 7,000 works of art. Kamstra's team creates encounters with art for patients, hospital staff, students, and visitors along the hospital's corridors, public spaces, and clinical rooms. The art department at Amsterdam UMC participates in collaborative research to explore the impact of art within the context of a major urban teaching hospital. Kamstra discussed the work of artist Nieke Koek who through conversations with patients and staff at the hospital’s rehabilitation clinic created an automata of a leg. The leg was intended to help individuals discuss and represent the experience of pain. “We believe that artists can give a different insight on specific medical questions, which can be of help to medical research,” Kamstra said.

**Jasminko Halilovic**, Founder and Managing Director of the War Childhood Museum in Sarajevo, Bosnia and Herzegovina, discussed the process of creating and sharing a collection focused exclusively on childhoods that have been affected by war. Halilovic emphasized how the museum, with its ability to respect, care for, and amplify an individual's story can contribute to both the individual and collective healing of pain and trauma. “The process of giving a personal object to the museum collection is not a mere act of donation,” Halillovic said. “This is a long-term connection, which is developed between people and museum. And this [connection] then transcends to the interactions with visitors.” In addition to a permanent museum and traveling exhibitions, the War Childhood Museum hosts workshops for teachers and parents on how to discuss the sensitive topic of conflict with children at home and in the classroom.

**Ine Gevers**, Founding Director of the Niet Normaal Foundation, Institute for Inclusive Innovation in Utrecht, The Netherlands, highlighted several of her exhibitions that focused on radical inclusivity and the dichotomy of pain and pleasure. “We really make a mistake in thinking that people with disabilities or chronic illness are [the] minority. They are actually a majority,” Gevers said. Her most recent exhibition, *Come Alive*, is a large-scale immersive experience that explores eroticism as a creative energy that can help individuals to reconnect with themselves and others in precarious times. The exhibit invites attendees to reflect on the redistribution of sensual love to all who need it as well as the use of pleasure to release pain.

## Arts experiences and practices

### How do we make art that reduces the burden of pain?

**Jeroen Lutters**, Professor at the ArtEZ University of the Arts in Arnhem, The Netherlands, challenged participants to consider the spectator as artist. Lutters presented an overview of Arts Based Learning (ABL), a method for learning from art that questions the duality between receiving and making an aesthetic experience. ABL asks the spectator to begin with a question of personal relevance, and then to bring that question into an extended dialogue with an object of art. The individual then enters into “possible worlds,” as Lutters explains, allowing the dialogue between art and spectator to generate responses to the original question. This process positions the spectator viewer as co-creator, and not consumer, of art. Lutters offered conference attendees the hypothetical of an individual who could embark on this process with the question, *how can I get rid of this pain?*

Designer and Artist, **Nienke Helder**, Eindhoven, The Netherlands, then spoke about her project, “Sexual Healing,” in which she developed several objects that invite women to explore their bodies and sexuality in a safe and non-clinical way. She described her design process, which involved partnering with medical experts and women seeking help for “sexual dysfunctions” such as painful intercourse, shame, problems with penetration, difficulties with getting in the “mood” or orgasms. Helder emphasized the need for, and challenges with, evaluating the impact of her work in the world. “After all,” Helder explains, “It's through this public engagement that we will be able to translate new knowledge into tangible experiences for those who eventually need them.”

Social designer, **Joost van Wijmen**, based in Hertogenbosch, The Netherlands, discussed his project ENCOUNTER #6. Van Wijmen invited individuals with bodily scars to partner with him and his creative team to make silk embroidered reproductions of those scars. These testaments to the changing body reveal the potential for even the most painful and difficult transformations to be expressions of beauty and meaning. ENCOUNTER #6 is displayed in a mobile exhibition that travels to libraries, museums, and hospitals among other locations. The project allows participants to enter into a deep conversation about their scar and their changing body, while also reframing these processes as a work of art. Viewers engage with such universal themes as intimacy, loss, and vulnerability. Van Wijmen says, “I don't solve problems. I listen mainly and provide a space for both funny stories, or sometimes success, but also discomfort. The goal of ENCOUNTER is sharing personal experiences … I ask participants or an audience to place themselves in the shoes of the other.”

**Mohsin Mohi-Ud-Din**, Founder and CEO of #MeWeInternational, which is headquartered in Georgia, United States, but works in 15 countries to provide communications tools that enable individuals to unlock their agency, reframe their narratives, and author the future, invited the audience to understand communication as foundational to art and health. Mohi-Ud-Din called for considering “words as living things that actually reshape the brain, reshape your nervous system, reshape how you view yourself.” MeWeInternational’s work is a powerful example of the impact that narrative art can have on pain across multiple contexts with program evaluations demonstrating improved communication skills, emotion regulation, goal-setting, problem-solving, perspective taking, and creative publishing opportunities. However, Mohi-Ud-Din also shared critical insights into the potential harm that can come from measuring the lived experience of people sharing personal experiences where, “The process of monitoring, evaluation, and data gathering retraumatized the communities that this data was meant to serve.” Mohi-Ud-Din called for balancing community control of language and process with scientific integrity.

## Research and creative scholarship

### How do we evaluate art intended to reduce the burden of pain?

The University of California, Davis (UCD) is involved in a number of studies exploring the public health potential of museums and arts spaces to address the burden of persistent pain. **Ian Koebner**, Director of Integrative Pain Management at UCD and Cultural Agents Fellow at Harvard University, discussed his partnership with the Crocker Art Museum in Sacramento, California to create and evaluate museum-based programs for individuals who self-identify as living with persistent pain. Their initial studies focused on the feasibility of the partnership from an organizational perspective (6) and from the perspective of individuals living with persistent pain (7). UCD is currently conducting the first ever randomized controlled trial of museum- and virtual-museum-based programs for individuals who self-identify as living with persistent pain (8).

Museum-based interventions can be conceptualized as complex in that they involve multiple and interacting factors (9). For example, the effect of the art in any museum-based program will interact with the group-dynamics, as well as the spatial context of the museum itself, to create a total effect. Disaggregating these factors in an effort to demonstrate causality can be difficult. **Jorge Peña**, a Professor in the Department of Communication at UC Davis, discussed a study that he co-leads with Koebner, to explore the specific effects of art and social connection in a virtual-museum model. Individuals living with persistent pain are randomized to a virtual museum gallery with or without art and with or without a social connection prime to test the separate and joint effects of these two factors—art and social connection (10).

**Sarah Herrera**, Assistant Director of Business Intelligence at the Mondavi Center for the Performing Arts at UCD shared how the Mondavi Center has initiated a program to learn more about the health of the people who visit the Center, as well as the degree to which they feel they belong at the Center. Herrera noted that the cultural sector has many audience development efforts, however less work is done to investigate how those audiences feel in cultural spaces, and how those feelings may differ among subpopulations. “Our hope is through understanding the experiences of those living with chronic pain that we may create experiences where they feel like they belong more in our spaces,” Herrera said. Herrera also provided insight into how museums and other art spaces might expand their diversity, equity, inclusion, and accessibility efforts by centering the construct of belongingness.

**Olivier Beauchet**, Professor and Director of AgeTEQ Laboratory at the University of Montreal, concluded the Research and Creative Scholarship section of the conference with a discussion of his work examining the effects of museum-based participatory arts programs on frailty, health, and socialization among the elderly. Beauchet first discussed a study with the Montreal Museum of Fine Art (MMFA) to explore the effects of a participatory art-based activity called “Thursdays at the Museum” for older adults (11). Following this study, the Fuji Museum, Tokyo, Japan, joined the partnership between the MMFA and the AgeTeQ laboratory in a bicentre RCT that enrolled 228 community-dwelling older adults to receive either a 12-week participatory art-based program or the control condition, which involved no art-based interventions over the study period. Well-being and quality of life improved significantly in the intervention group compared to the control group, while mixed results were observed with frailty (12).

## Conclusion

The 21st century ushered in two seemingly disparate truths that the *Analgesic Museum* conference sought to bridge: the social dimension of persistent pain is inadequately addressed in the current biomedical model of care (2, 13) and the arts may be an unlikely, yet valuable public health partner (14). The day of dialogue offered a creative response to the difficult and important question of if, and how, the arts can relieve pain. An international interdisciplinary group of individuals with the lived experience of persistent pain, policy makers, funders, scientists, curators, museum and healthcare professionals, and artists came together to share their experiences at the intersection of art and pain management practices. Panelists emphasized the potential benefits of arts-engagement on pain, from mitigation of pain and pain-related outcomes for the individual to facilitating education and compassionate understanding for society. A common theme among the diverse and myriad project examples shared by panelists was the importance of creating opportunities for individuals to author their experience of pain and to engage in dialogue about those experiences. Panelists also stressed the challenges associated with developing arts-based experiences that target the reduction of persistent pain. Examples of challenges include intervention design, appropriate evaluation methods, and partnering across divides such as epistemological perspective (e.g., positivism vs. constructivism), discipline (e.g., the “arts” vs. the “sciences”) and social position (e.g., “able” vs. “disabled” bodies).

The Analgesic Museum conference was a critical if first step in the establishment of a framework for exploring the aesthetics and impact of museum engagement to reduce the burden of persistent pain. This manuscript aims not only to highlight and amplify specific projects at the intersection of art and pain management practices, but also to showcase the conference itself as a mechanism for network building in this undeveloped topic area. The conference's themes—Exhibition Development, Arts Experiences and Practices, and Research and Creative Scholarship—offer domains of practice for developing the field of art and pain management. Our hope is that the conference will serve as a template for additional convenings that center other regions of the world, with the ultimate goal of creating a global interdisciplinary network dedicated to the intersection of art and pain management practices.

## Data Availability

Conference presentations can be found at https://health.ucdavis.edu/pain/acupuncture/CrockerArtRx.html. Further inquiries can be directed to the corresponding author/s.
